# Reply to: “Historical pursuits of the language pathway hypothesis of schizophrenia”

**DOI:** 10.1038/s41537-021-00183-y

**Published:** 2021-11-09

**Authors:** Lena Palaniyappan, Jingnan Du, Jie Zhang, Jianfeng Feng

**Affiliations:** 1 Department of Psychiatry and Robarts Research Institute, University of Western Ontario, London, Ontario Canada; 2 Lawson Health Research Institute, London, Ontario Canada; 3 Institute of Science and Technology for Brain-Inspired Intelligence, Fudan University, Shanghai, China; 4 Shanghai Key Laboratory of Psychotic Disorders, Shanghai Mental Health Center, Shanghai Jiao Tong University School of Medicine, 200030 Shanghai, China; 5 Key Laboratory of Computational Neuroscience and Brain Inspired Intelligence (Fudan University), Ministry of Education, Shanghai, China; 6 Department of Computer Science, University of Warwick, Coventry, CV4 7AL UK

**Keywords:** Schizophrenia, Biomarkers

**replying to** L.E. DeLisi *npj Schizophrenia* 10.1038/s41537-021-00182-z (2021)

We appreciate the comments by Dr. DeLisi^1^ regarding our study investigating the genetic determinants of language-network dysconnectivity in early-stage schizophrenia^2^. In her comments, Dr. DeLisi highlights the missing historical context of the focus of our study and the lack of evidence linking our choice of language-related genes to the elevated genetic risk for schizophrenia. We concur with both of her observations and discuss how we can make further progress from the matters arising in this field of inquiry.

As pointed out by Dr. DeLisi, the three major contributions to our current thinking in the field of language and psychosis are Crow’s theory on evolutionary origins, Chaika’s linguistic exposition, and the large body of imaging work led by Dr. DeLisi. We fully agree that the association among language, schizophrenia and its genetics as proposed by Crow has been examined much earlier in several other works (see DeLisi et al.^3^ and other studies^4–8^). More recently, a comparative connectomics study has established that schizophrenia-related dysconnectivity occurs in regions displaying human-specific connections not seen in chimpanzees^9^, adding support to Crow’s notion that schizophrenia may be a price we pay for human evolution. Nevertheless, testing the genetic basis of the putative link between the *evolution of language* per se and the features of schizophrenia has been hitherto elusive. Thus, a direct association between genes that supposedly influence ‘language-readiness’ in humans and network-level dysconnectivity in schizophrenia remained unexamined prior to our study^2^.

Dr. DeLisi cites several works that focussed on core language network as the substrate of the genetic risk of schizophrenia; in contrast, in our work we remained agnostic to the patterns of dysconnectivity that can be expected across the various stages of schizophrenia. In other words, we did not select the language regions a priori to study their dysconnectivity patterns; instead, we employed a “discovery” approach and conducted a brain-wide search. This agnostic stance comes from the large body of resting-state connectivity literature in schizophrenia and in genetic high-risk states where language networks have not emerged as the most affected modules (see Dong et al.^10^, Del Fabro et al.^11^, and also Liloia et al.^12^). A purview of studies that specifically examine disruptions in overt linguistic aspects of schizophrenia (also called formal thought disorders), indicate that brain networks other than the core language network also play a crucial role in the clinical expression of schizophrenia^13^. There may be many methodological reasons for this observed lack of language-network primacy in fMRI literature that is beyond the scope of the current discussion. We believe some of the inconsistencies may indeed emerge from the effects of disease progression on the patterns of dysconnectivity; separating patients with short and long duration of illness may provide more clarity. As a result, we undertook a whole-brain voxel-wise search to locate the spatial distribution of dysconnectivity separately in the short and long duration groups in a schizophrenia dataset consisting of 138 drug-naive first-episode schizophrenia patients and 112 healthy controls^2,14^. Our approach can reduce the false-positive rate and increase statistical power by appropriately utilizing the spatial information of fMRI data^14^. In our previous publications in collaboration with Crow, we have pursued the same brain-wide search approach^15,16^, and demonstrated that patients with first-episode schizophrenia have functional dysconnectivity most prominently in the left inferior frontal gyrus (including Broca’s area)^15^, and male patients in particular exhibit a predominantly left-lateralized pattern of aberrant connectivity with a focus on Broca’s area^16^. Given the lack of a priori selection language networks in our study^2^, we did not bring up the large body of fMRI and structural literature referred to by Dr. DeLisi that focuses on these networks.

In contrast to our lack of a priori selection of language-network seeds for the fMRI, we specifically chose a set of *language-readiness* genes for our pathway-specific polygenic risk scores. This was directly motivated by Crow’s notion of linguistic primacy in schizophrenia and its connection with human evolution. Our selected candidate genes for language-readiness overlap with schizophrenia as well as brain development as listed by Murphy&Benitez-Burraco in their review^17^. The motivation for clustering these genes has been expounded in detail elsewhere^18–20^. Our interest in FOXP2 was ignited by the various inherited and de novo variations in this gene noted in children with linguistic developmental disorders^21^ and the likelihood of its recent evolutionary selection (though this notion has come under scrutiny; see refs. ^22,23^). We concur with Dr. DeLisi that “the genetics of schizophrenia remain elusive and is likely to be highly heterogeneous”. She is right to point out that FOXP2 pathway has not been implicated in large-scale studies seeking genome-wide associations or copy number variations as susceptibility markers for schizophrenia.

While the linguistic features of schizophrenia (often grouped as “formal thought disorders”) are notably familial, no single genetic pathway has been consistently associated with the linguistic readouts that typify this illness (see Nicodemus et al.^24^ and Nestsiarovich et al.^25^ for preliminary associations reported in this regard). It is possible that the genetic factors that influence linguistic features and the language-network architecture in schizophrenia are distinct from those that increase the susceptibility of schizophrenia per se. For example, subtle features of formal thought disorder can occur in otherwise healthy biological parents of patients^26^. In Finnish Family Adoption studies, a family history of schizophrenia in biological parents did not confer an increased risk of formal thought disorders among the children living with adopted parents^27^. On the other hand, the presence of schizophrenia increases the risk of subtle formal thought disorders among family members^28^. Thus, we do not consider it necessary that a genetic pathway related to a specific feature such as functional dysconnectivity or formal thought disorder of schizophrenia should also relate to overall susceptibility to this complex polygenic illness. The fact that FOXP2 variations do not confer an elevated risk of schizophrenia, does not diminish the observation that its variations relate to the degree of dysconnectivity in the early stages of schizophrenia (for further discussion, see ref. ^29^).

We and Dr. DeLisi agree on the notion of linguistic primacy in schizophrenia; we only differ in the roads we have chosen to travel. An impressively large body of work has tested if the genetic risk for schizophrenia primarily affects the language-network connectivity; in contrast, our modest work examined if the pattern of distributed dysconnectivity in the early stages of this illness relates to a specific genetic pathway of language readiness (FOXP2). Unlike the prior works cited by Dr. DeLisi, we do not test if the overall genetic susceptibility to schizophrenia converges on the language network per se. Instead, we report a possible genetic basis for the dysconnectivity of language-relevant regions early in this illness. The FOXP2 pathway does not appear to influence the pattern of dysconnectivity in the later stages of the illness, or the dysconnectivity of regions other than the left inferior frontal gyrus in the early stages. We recognize that the original paper, especially the content related to the language pathway hypothesis in our introduction, did not give a full overview of the valuable preceding work that led us here^1^. Nevertheless, our choice of genetic targets centered on language-readiness remains close to Crow’s original evolutionary notion of schizophrenia.

Our observation that a polygenic language-readiness pathway has a statistical association with Broca’s area dysconnectivity in the early stages of schizophrenia, if replicated by others, may provide one piece of the puzzle that connects the evolution of language with schizophrenia. What value does the solution of this puzzle holds to the labyrinth of mechanistic origins of schizophrenia, is a matter that requires deeper consideration. We believe sustained multidisciplinary and international efforts are required to examine language in psychosis; in this regard, we support the new initiative ‘DISCOURSE in Psychosis’ (https://discourseinpsychosis.org/) that aims to tackle this in a collaborative manner.

## Data Availability

The datasets generated during the current study are not publicly available due to ethical codes for this study but are available from the corresponding author on reasonable request with the approval of The Research Ethics Committee of the Shanghai Jiao Tong University School of Medicine.

## Code availability

No new codes were generated for this response. The BWAS: Voxel-level connectome-wide association studies code is publicly available at https://github.com/weikanggong/BWAS. The code used for the original study by Du et al. is publicly available at the following URL: https://osf.io/2zaqv/.
